# Editorial: Deepening consciousness: what phenomenology, yogic, and Buddhist meditation can contribute from a psychological perspective

**DOI:** 10.3389/fpsyg.2026.1815107

**Published:** 2026-03-18

**Authors:** Christopher Gutland, Selenia di Fronso, Stephan Schleim

**Affiliations:** 1 Institute of Foreign Philosophy, College of Philosophy, Zhejiang University, Hangzhou, China; 2 Department of Theoretical and Applied Sciences, eCampus University, Novedrate, CO, Italy; 3 Theory and History of Psychology, Faculty of Behavioral and Social Sciences, Heymans Institute for Psychological Research, University of Groningen, Groningen, Netherlands

**Keywords:** Buddhism, consciousness, first-person methods, meditation, methodology, phenomenology, psychology, yoga

Yogic and Buddhist meditation, as well as phenomenology, share the goal of deepening conscious experience. Yet they pursue it under different “methodological axioms.” In many yogic and Buddhist contexts, the cultivation of attention, insight, and ethical orientation is organized along pathways of practice in which intermediate experiences are typically articulated as markers of training, diagnostics of progress, and orientations toward what comes next. Phenomenology, by contrast, is classically animated by the ambition to describe consciousness with intersubjective rigor: to disclose what is always already there in everyday experience but habitually overlooked. It does so through disciplined reflection and the phenomenological “bracketing” of everyday assumptions (epoché/reduction) to attend to how experience presents itself. This descriptive ambition is accompanied by an abiding worry: that the very shift of attention required for description may already transform the phenomenon, risking distortion.

This Research Topic views this tension as productive, not paralyzing. If meditative practice can durably transform experience, and if phenomenology can refine how conscious experience becomes describable, then the interface between transformation and description becomes a shared research problem—philosophical, psychological, and methodological. Addressing it requires the traditions to move beyond conventional self-descriptions. Phenomenology is challenged to ask whether its methods can be applied to (or must be retooled for) altered and cultivated modes of consciousness, including healthier and more aware forms of lived experience. Meditative traditions, conversely, are challenged to clarify the extent to which their accounts of altered states—often embedded in lineage-specific metaphysics, soteriology, and technical vocabularies—can be rendered comparable without either flattening what matters or smuggling doctrine into description.

A central aim, therefore, is conceptual and terminological: the articulation of an intersubjective and coherent lexicon for transformations of consciousness and conscious experience—one that is neither merely tradition-bound nor reduced too quickly to third-person neurobiological or psychometric descriptions. This is where psychology enters. Contemporary psychology and neuroscience have made meditation research widely legible from a third-person perspective, but the most distinctive claims of both phenomenology and contemplative lineages are irreducibly first-person. The question is not whether first-person methods “count,” but what methodological tools—training regimes, interview practices, second-person elicitation, disciplined reflection, triangulation with behavioral and physiological measures—can secure rigor without evacuating subjectivity. The contributions collected here approach these shared tasks from complementary angles as follows:

Milicevic et al. have contributed a methodological article on combining phenomenological perspectives from the first- and second-person viewpoints with third-person electroencephalography (EEG) data to investigate mindfulness and consciousness. Their mixed-methods design is based on Buddhist psychology and integrates verbal reports and neuroscientific data from researchers and contemplative participants.

Schleim examines the compatibility of Western and yogic psychology, as described in an old treatise used worldwide in yoga teacher trainings. He emphasizes the epistemological prerequisites for concept formation in both fields and problematizes the concept of “yogic science” particularly in light of claims about parapsychological phenomena.

Wang discusses how different Buddhist meditation “pathways” (Samatha, Vipassana, Metta) foster specific metacognitive insights, emotional regulation, and self-inquiry, leading to transformative shifts in consciousness and offering a practical comparative framework for psychological research. Evidence (brain network changes) shows diverse effects on attention and emotional patterns. Notwithstanding methodological challenges, Wang advocates interdisciplinary, culturally sensitive research approaches.

Ramirez-Duran et al. used Reflexive Thematic Analysis to explore how Ashtanga practitioners experience yoga. The analysis revealed that yoga is experienced as an embodied practice for health, personal inquiry, and spiritual development. In conclusion, while daily practice integrates these principles, in their sample, yoga does not appear to be primarily physical or stress-focused, and its philosophy provides guidance on consciousness.

Wittmann et al. link the Eastern concept of mindfulness with the Stoic idea of self-regulation within the context of modern psychology and neuroscience. They argue that this enables a constructive understanding of free will through the expression of second-order volitional actions over time, and thus of the self as a self-determined, free agent.

Arora reframes the “hard problem” transpersonally: consciousness is ontologically primary, not an emergent neural product. Drawing on non-dual traditions (Advaita Vedanta, Tibetan Buddhism) and transpersonal theory, she critiques materialist reductionism and defends first-person and participatory ways of knowing. The article sharpens this topic's stakes: what counts as evidence in consciousness research?

Gutland and Liu reconstruct Liangkang Ni's reading of Husserl's reflections on Buddhism and phenomenology, as well as Ni's mapping of phenomenology onto Yogācāra accounts of consciousness. The paper identifies parallels and divergences between the traditions to sharpen a shared conceptualization and methodological agenda for first-person research in psychological contexts.

Loncar and Fiskum theorize “detached self-observation”—non-identification with thoughts and feelings—as a culturally scaffolded developmental competence. This builds a bridge between phenomenological stance-taking (epoché-like distance) and meditation-driven transformation. Integrating cultural-historical psychology, embodied phenomenology, dynamical-systems “attractor” change, and virtue ethics, they portray benefits and risks, urging context-sensitive research.

Ren diagnoses a mismatch in the study of self-consciousness: quantitative psychology operationalizes it as a measurable trait, while the Husserlian reduction treats it as a prereflective dimension of experience. Rather than rejecting measurement, Ren argues phenomenology clarifies the meaning and limits of constructed units and scores, enabling more rigorous first-person science.

Taken together, these articles do not merely juxtapose “East” and “West,” or practice and theory. They foreground a shared methodological frontier: how cultivated transformations of experience can be described without being domesticated, and how description can become scientifically tractable without being reduced—opening space for a psychology of deepening consciousness that is empirically rigorous *and* intersubjectively anchored in first-person methods and shared terms.

